# Gene Expression Signatures of Smoking and Acute Myocardial Infarction: A Blood Transcriptome Analysis

**DOI:** 10.1155/mi/2431090

**Published:** 2025-01-15

**Authors:** Fang-Fang Liu, Yi-Xuan Yan, Hong-Feng Zhang, Ke Li

**Affiliations:** ^1^Department of Pathology, The Central Hospital of Wuhan, Tongji Medical College, Huazhong University of Science and Technology, Wuhan 430014, China; ^2^Department of Blood Transfusion, Tongji Hospital, Tongji Medical College, Huazhong University of Science and Technology, Wuhan 430030, China

**Keywords:** acute myocardial infarction, blood transcriptome, cardiovascular biomarkers, differential gene expression, smoking

## Abstract

**Background:** Tobacco smoke is known to contain numerous harmful chemicals, and epidemiological evidence has firmly established smoking as a potent risk factor for hypertension and myocardial infarction (MI). However, the precise mechanisms by which smoking contributes to cardiovascular disease are not fully understood. The aim of this study is to identify common molecular signatures in blood that link smoking to acute MI (AMI).

**Methods:** We extracted transcriptome data from seven blood microarray datasets in the Gene Expression Omnibus (GEO) database, encompassing a total of 403 patients. Employing both individual dataset analysis and a combined meta-analysis approach, we conducted a thorough examination of blood transcriptome profiles associated with AMI and smoking, uncovering numerous differentially expressed genes (DEGs).

**Results:** Functional enrichment analysis indicated that DEGs associated with AMI and smoking were significantly enriched in overlapping biological processes, such as immune response and inflammation. Moreover, three genes—PTGDR, PYHIN1, and PRSS23—were consistently altered in both conditions and were validated as dysregulated in AMI using an independent GEO dataset. Furthermore, quantitative real-time reverse transcription polymerase chain reaction (qRT-PCR) validation further confirmed the differential expression of PYHIN1 and PRSS23 in AMI patients.

**Conclusions:** Our findings suggest that gene expression changes induced by smoking in blood may contribute to the heightened risk of AMI. These identified genes are likely to play critical roles in the pathogenesis of AMI. Given the accessibility of peripheral blood samples, the expression levels of these genes could potentially serve as biomarkers for assessing cardiovascular health, particularly in individuals with a history of long-term exposure to cigarette smoke.

## 1. Introduction

Acute myocardial infarction (AMI) is one of the most common diseases all over the world. AMI occurs when blood stops flowing normally to a part of the heart, and the heart muscle is injured due to lack of oxygen. More than 800,000 people worldwide experience AMI each year, 27% of them die [1]. AMI is most commonly due to the blockage or occlusion of a coronary artery following the rupture of a vulnerable atherosclerotic plaque, an unstable buildup of white blood cells, cholesterol, and fat in the arterial wall [2].

Smoking is a well-known reversible risk factor of AMI. Compared with nonsmokers, smokers had a relative risk of MI of 2.24 (female; range 1.85–2.71) and 1.43 (male; range 1.26–1.62) [3]. Studies have shown that smoking rates in “young” MI patients can be as high as 89% [4]. It has been reported that about 33% of cardiovascular-related deaths are attributable to smoking [5]. Harmful ingredients of cigarettes are absorbed first in the lungs, then into the blood, and spread throughout the body. It has been found smoking induces gene expression changes in blood cells [6]. The most common infarction mechanism in smokers seems to be atherosclerosis caused by chronic inflammation, in which blood immune cells play vital roles [7, 8]. The profile of white blood cells changes drastically during AMI [9]. AMI patients also show broadly altered gene expression in peripheral blood [10–12]. Hence, gene expression changes associated with chronic inflammation in the blood caused by smoking may be implicated in the increased risk of AMI due to cigarette smoking.

In the present study, we provided a comprehensive blood transcription signature of AMI and smoking. Gene expression markers of AMI and smoking in peripheral blood were uncovered. The commonly altered genes between AMI and smoking may also be potential indicators of cardiovascular health.

## 2. Materials and Methods

### 2.1. Patients, Samples, Data Extraction, and Study Design

**Figure 1:**
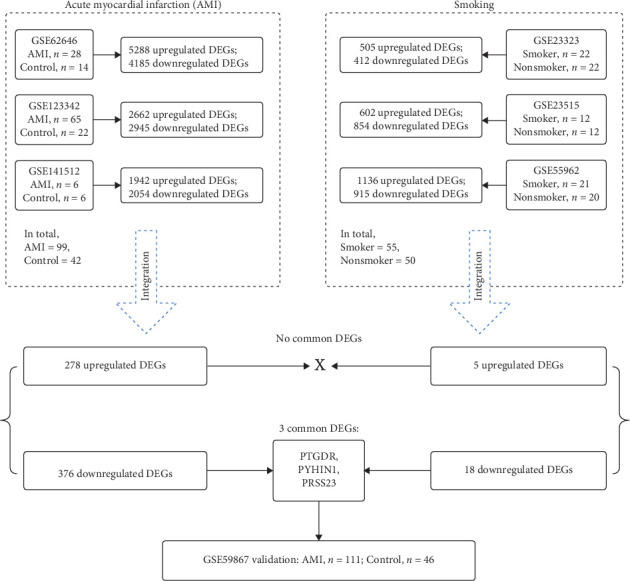
The flowchart of the present study. AMI, acute myocardial infarction; DEGs, differentially expressed genes.

Gene expression data used in the present study were extracted from four blood transcriptome datasets about AMI and three blood transcriptome datasets about smoking, containing 403 patients in total. These datasets were available in the public Gene Expression Omnibus (GEO) database. For the four AMI datasets, the accession numbers in the GEO database were GSE141512, GSE62646, GSE123342, and GSE59867, respectively. GSE141512, GSE62646, and GSE123342 datasets were used for Limma analysis, containing a total of 99 AMI blood samples (1st day of AMI) and 42 control blood samples. The GSE59867 dataset was used for the final validation of the identified hub genes, comprising 111 AMI blood samples (1st day of AMI) and 46 control blood samples. For the three smoking datasets, the accession numbers in the GEO database were GSE23323, GSE23515, and GSE55962, containing a total of 55 smoking and 50 nonsmoking blood samples. All the demographic and clinical data of the study sample can be retrieved from previous research [10–14]. The analysis procedure of the present study and sample details about each GEO dataset are shown in Figure 1 and Supporting Information 1: Table S1. For the quantitative real-time reverse transcription polymerase chain reaction (qRT-PCR) validation, we enrolled five patients diagnosed with AMI on their first day of symptoms, admitted to our hospital between November 28, 2024, and December 16, 2024. The diagnosis was confirmed based on clinical symptoms, ECG changes indicative of ischemia, and elevated cardiac troponin levels. The data for these patients were all derived from their medical records, and no follow-up visits were conducted post-discharge. The Ethics Committee of Tongji Hospital approved this study with a waiver of informed consent. The major characteristics of these patients are shown in Table 1. All these patients underwent blood typing in the Department of Blood Transfusion on the first day of their admission (1st day of AMI), and we utilized the discarded blood samples from these tests for qRT-PCR analysis. In addition, we also enrolled five age-matched normal individuals; they had no history of cardiovascular diseases, diabetes, or any major health issues, and their routine health examinations were all within the normal range. Peripheral blood mononuclear cells (PBMCs) were isolated from these samples using density gradient centrifugation. RNA extraction was performed, and the concentration and purity of the RNA were assessed using an ultraviolet spectrophotometer. Quantitative PCR (qPCR) was conducted using the StepOne Plus real-time PCR system (ABI, Carlsbad, CA, USA), with GAPDH as the internal control. The relative gene expression was determined by the 2^−*ΔΔ*CT^ method, and the primers for each gene tested are as follows: GAPDH (F: 5′-CTCTGCTCCTCCTGTTCGAC-3′; R: 5′-ACGACCAAATCCGTTGACTC-3′), PTGDR (F: 5′-CTGGGCAAGTGCCTCCTAAG-3′; R: 5′-CAACGAGTTGTCCAATGCGG-3′), PYHIN1 (F: 5′-CCAAGCAACCGTCTCACAG-3′; R: 5′-GCCGAGTCTGCTCTTTGGA-3′), and PRSS23 (F: 5′-TGTGCTGTTGGGCAAGTGAG-3′; R: 5′-AGTTCCCTTATGACACTGGGG-3′), SLAMF7 (F: 5′-ACCCTCATCTATATCCTTTGGCA-3′; R: 5′-CACCAACGGAACCGACCAG-3′).

### 2.2. Differential Expression Analysis

The Limma package [15] in the R/Bioconductor (version 3.5.1) software and the GEO2R tool were utilized to obtain differentially expressed genes (DEGs) from the above datasets. GEO2R is an interactive web tool officially provided by the NCBI GEO database (https://www.ncbi.nlm.nih.gov/geo/geo2r) [16]. The cutoff criteria for DEGs were set as the adjusted *p*-value <0.05 (the *p*-value is adjusted using the Benjamini–Hochberg method). Volcano plots were used to display the distribution of DEGs in each dataset. Venn diagrams and upset plots were used to show the integration of DEGs in different datasets. The upset plots were generated by the UpsetR package in R software [17]. Considering pooling datasets before analysis will increase the sample size and statistical power, we also combined analysis for the three AMI datasets, GSE141512, GSE62646, and GSE123342. Since the three AMI datasets used two different microarray platforms, they cannot be pooled together by simply removing batch effects by “limma” [15] or “sva” packages [18] in R. We chose a “meta-analysis method” to combine the three AMI datasets for removing batch effects. This method is suitable for analyzing multiple datasets with different platforms. The process of combining analysis was conducted in ImaGEO, a web-based platform [19]. Briefly, after integrating all three GEO datasets (GSE141512, GSE62646, and GSE123342), ImaGEO performed background correction, normalization, batch effect correction, and applied initial differential expression analysis. This meta-analysis method introduced a parameter termed “effect size,” which is considered combinable and comparable across different expression datasets. Analysis parameters in ImaGEO were set as: “effect size estimation model” = “Random effect model;” “Allowed missing values (%)” = “10;” “Adjusted *p*-value threshold” = “0.05.” This method will output a *Z* value for each DEG. This *Z* value is a test statistic for *Z*-tests, which measure the difference between an observed statistic, the gene expression levels, and its hypothesized population parameter in units of the standard deviation. In other words, this *Z* value can reflect the direction of gene expression changes in the combined dataset (positive or negative fold change, indicating upregulated or downregulated, respectively).

### 2.3. Functional Enrichment Analysis

We performed Gene Ontology (GO) enrichment and pathway analysis for identified DEGs in AMI and smokers, respectively. GO analysis, including biological process (GO_BP), molecular function (GO_MF), and cellular component (GO_CC), was performed using DAVID v6.8 (https://david.ncifcrf.gov/home.jsp) [20]; pathway analysis was conducted in Reactome online database (Version 77:09 June 2021. https://reactome.org/) [21]. We also performed GSEA for the 1698 DEGs generated by the meta-analysis method from the three AMI datasets, GSE141512, GSE62646, and GSE123342. GSEA was conducted using “clusterProfiler” and “ReactomePA” packages in R [22, 23]. The input DEG list was ranked by the *Z* value of each DEG. The cutoff *p*-value was set as 0.05, and the *p*-value was adjusted by the “Benjamini–Hochberg” method.

## 3. Results

### 3.1. Analysis of DEGs in AMI

**Figure 2:**
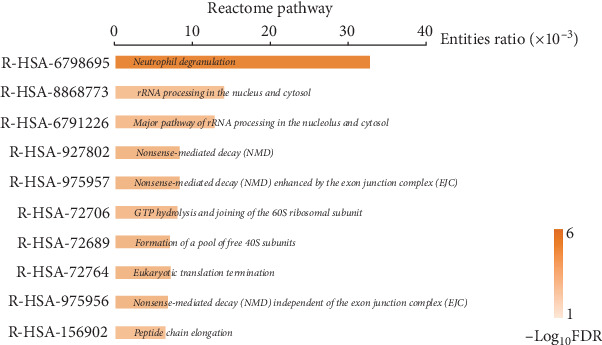
Reactome pathway analysis for DEGs identified in AMI. The length of the bar represents the significance of an enriched term (entities ratio). The brightness of the bar represents the negative log_10_FDR. The brighter the color is, the more significant that term is. AMI, acute myocardial infarction; DEGs, differentially expressed genes.

**Figure 3:**
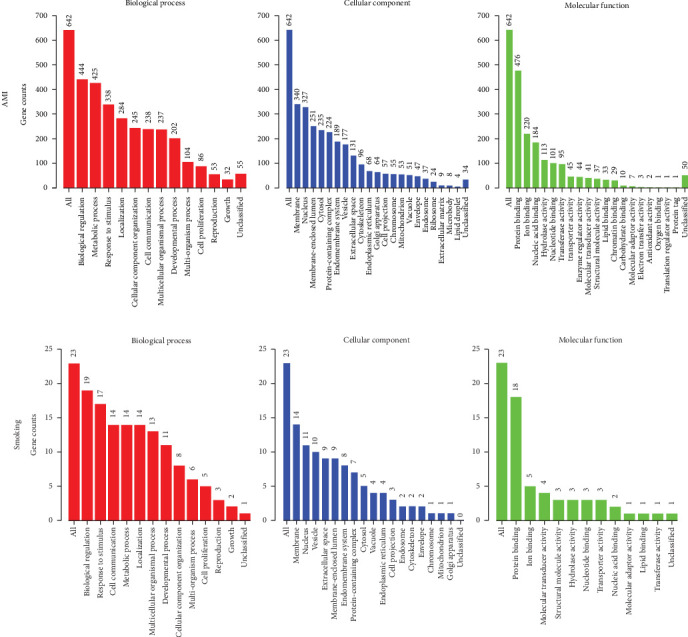
GO analysis for DEGs identified in AMI and smoking. (A) GO results for DEGs in AMI. (B) GO results for DEGs in smoking. Each column represents an enriched GO term. The number at the top of each column shows the total number of DEGs that fall into that term. AMI, acute myocardial infarction; DEGs, differentially expressed genes; GO, Gene Ontology.

To explore gene expression changes in blood during AMI, we retrieved the gene expression data from the NCBI GEO database. Three GEO datasets were selected for DEG identification, as mentioned in Section 2. DEG results were visualized with volcano plots (Supporting Information 2: Figure S1A–C). In the GSE123342 dataset, 2662 upregulated and 2945 downregulated genes were identified (Supporting Information 2: Figure S1A). In the GSE141512 dataset, 1942 upregulated and 2054 downregulated genes were obtained (Supporting Information 2: Figure S1B). In the GSE62646 dataset, we acquired 5288 upregulated and 4185 downregulated genes (Supporting Information 2: Figure S1C). The overlap of gene changes between the three datasets shown is with Venn diagrams. In total, 278 upregulated and 376 downregulated genes during AMI were identified (Supporting Information 3: Figure S2 and Supporting Information 4: Table S2). Pathway results showed these genes were mostly enriched in “Neutrophil degranulation” (Figure 2). As neutrophils are the first responders to inflammation, activation of the “Neutrophil degranulation” pathway reflects the initiation of inflammatory reaction after AMI. These DEGs were also enriched in several pathways linked with RNA or protein metabolism (Figure 2). This deregulation of RNA and protein metabolism may be related to leukocytosis during AMI, which may promote immune cell infiltration into the cardiac tissue. In addition, previous studies have reported that AMI affects the metabolism throughout the body [24]; thus, the RNA and protein metabolism dysfunction in peripheral blood is just the tip of the iceberg. GO analysis revealed that these identified genes were significantly related to several enriched terms, such as “biological regulation” for GO_BP, “membrane” for GO_CC, and “protein binding” for GO_MF (Figure 3A).

### 3.2. Analysis of DEGs in Smoking

**Figure 4:**
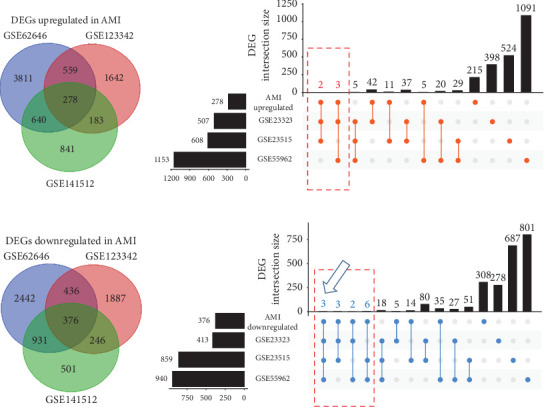
Venn diagrams and upset plots for DEGs identified in AMI and smoking. The Venn diagrams in (A) and (C) panels depict the integrated results in the three AMI datasets. The upset plots in (B) and (D) panels show the intersection of DEGs between AMI and the three smoking datasets. *AMI upregulated*, common DEGs upregulated in the three AMI datasets; *AMI downregulated*, common DEGs downregulated in the three AMI datasets. AMI, acute myocardial infarction; DEGs, differentially expressed genes.

We selected three associated GEO datasets to explore blood gene expression profiles in smoking. Overall, 507, 608, and 1153 upregulated genes were found in GSE23323, GSE23515, and GSE 55962 datasets, respectively (Supporting Information 2: Figure S1D–F); 413, 859, and 940 downregulated genes were also obtained in the GSE23323, GSE23515, and GSE55962 datasets, respectively (Supporting Information 2: Figure S1D–F). The upset plots showed there were five common upregulated and 18 common downregulated genes between the three smoking-associated datasets (Figure 4B, D and Supporting Information 5: Table S3). GO analysis of these smoking-related DEGs showed them to possess several same enriched GO terms compared with that of AMI (Figure 3B).

### 3.3. Comparisons of Gene Changes During AMI and Smoking

**Figure 5:**
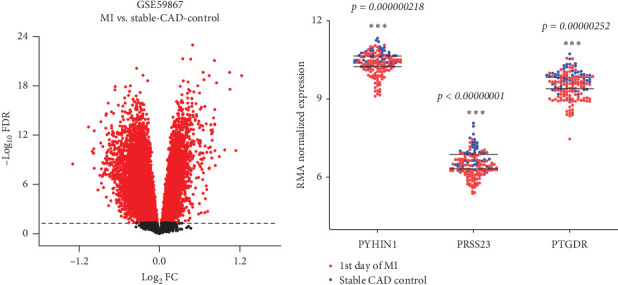
Validation of the three hub genes in the GSE59867 dataset. (A) Volcano plot for Limma results in the GSE59867 dataset. The *x*-axis represents log_2_ FC, and the *y*-axis indicates log_10_FDR. Each dot represents a gene that had detectable expression in both groups. Red dots represent genes that are significantly expressed in the AMI group compared with the stable-CAD-control group. FC, fold change; FDR, false discovery rate. (B) Relative expression of PYHIN1, PRSS23, and PTGDR in AMI patients and controls. *⁣*^*∗∗∗*^*p*  < 0.001 (*p*-value here were the adjust *p* value, FDR). AMI, acute myocardial infarction.

To find common DEGs between AMI and smoking, we cross-matched the genes altered during AMI to those altered in smoking and identified three candidate DEGs: PYHIN1, PRSS23, and PTGDR (Figure 4D and Table 2). To validate the blood expression level of the three genes in patients with AMI, we used an independent GEO dataset, GSE59867. Results showed that the expression levels of PYHIN1, PRSS23, and PTGDR were dramatically decreased in AMI patients than in stable CAD controls (Figure 5A,B). It is worth mentioning that expression levels of the three common DEGs in the peripheral blood were significantly decreased in patients with AMI or individuals after smoking (Figure 4D, indicated by the blue arrow). However, no common DEGs were found both upregulated in AMI and smoking conditions. This could be because our filtering strategy is too strict and requires merging all six datasets (three AMI datasets: GSE141512, GSE62646, and GSE123342; three smoking datasets: GSE23323, GSE23515, and GSE 55962). Nevertheless, as depicted in Figure 4, it is possible to identify more common DEGs, including upregulated and downregulated ones, if only one or two of the three smoking datasets are chosen instead of all three (Figure 4B,3, boxes with red dots). As a result, there would be a total of five upregulated and 14 downregulated common DEGs identified, and we also tended to believe that these common DEGs may be significant contributors to the increased risk of AMI due to cigarette smoking.

### 3.4. Integrated Meta-Analysis of Gene Expression Data From AMI Datasets and qRT-PCR Validation

**Figure 6:**
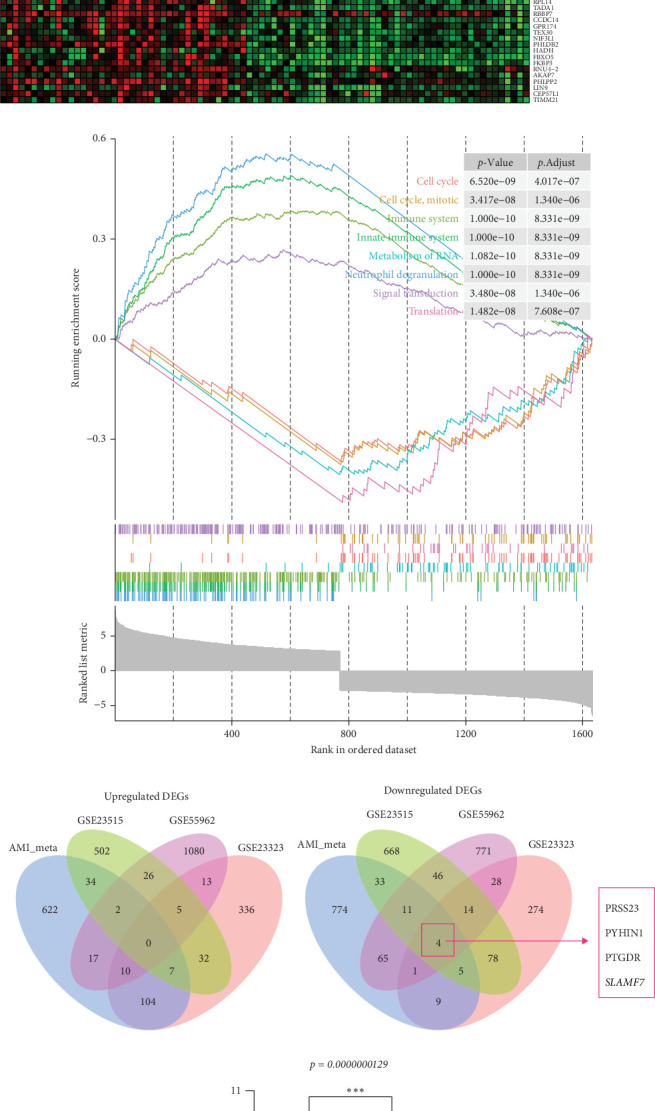
Results of meta-analysis pooling all three AMI datasets. (A) Heatmap of top 100 genes. The heatmap shows the expression of the top 100 most over and underexpressed genes in a set of samples of each AMI dataset. (B) GSEA for the 1698 DEGs identified by the meta-analysis method. The enrichment plot only shows the top eight enriched Reactome pathways. (C and D) Venn diagrams for DEGs were identified by the meta-analysis method (AMI datasets) and three smoking datasets. (E) Validation of SLAMF7 expression in the GSE59867 dataset. *⁣*^*∗∗∗*^*p*  < 0.001. AMI, acute myocardial infarction.

Although the above analysis using the intersection method (Venn diagrams) has identified common DEGs, to increase the sample size and statistical power, we further performed combined analysis (meta-analysis) for these three AMI GEO datasets, GSE141512, GSE62646, and GSE123342. In total, this meta-analysis method built a combined gene expression matrix with 141 samples (99 AMI samples and 42 control samples), which dramatically increased the sample size and meanwhile, also remained consistent with the total sample size of the above individual analysis. Through this matrix, we identified 1698 DEGs, including 796 upregulated and 902 downregulated (Figure 6A and Supporting Information 6: Table S4). Because in the above individual analysis, identified DEGs were input into the overrepresentation analysis (ORA), including GO and Reactome pathway analysis, to explore which GO or Reactome items they were enriched in. Here, similarly, we also conducted GSEA (Reactome pathway) for the 1698 DEGs. GSEA results showed that these DEGs were mostly enriched in pathways such as “Neutrophil degranulation,” “Innate Immune System,” “Immune System,” “Metabolism of RNA,” and so on (Figure 6B and Supporting Information 7: Table S5). Those results were generally consistent with the above individual analysis, suggesting the individual analysis method is feasible and reliable even though its relatively low sample size in every single dataset. Next, we intersected the 1698 DEGs with DEGs identified from smoking datasets. Four common hub genes were finally obtained (Figure 6C,D). Three of the four hub genes were the same as that from individual analysis, but the remaining one, SLAMF7, was newly identified and only obtained through the meta-analysis. We also validate the expression changes of SLAMF7 between the AMI and control group in the validation dataset GSE59867. The results showed that SLAMF7 was significantly underexpressed in the AMI group compared with the controls (Figure 6E). Furthermore, we performed qRT-PCR to validate the expression of the four hub genes in AMI patients. Results showed that the expression levels of PYHIN1 and PRSS23 were significantly downregulated (Supporting Information 3: Figure S2). In contrast, while there was a trend towards decreased expression for PTGDR and SLAMF7, these changes did not reach statistical significance. This absence of statistical significance may be attributed to the limited sample size. An expanded cohort is needed in the future to provide more definitive validation.

## 4. Discussion

Blood biomarkers are widely used in the diagnosis and management of cardiovascular disease, as blood sampling is minimally invasive, readily available, and repeatable. In the present study, we attempted to compare the blood transcriptome profile of AMI and smoking to explore common changes between them and to gain further insight into the potential molecular mechanism that how smoking contributes to an increased risk of AMI. Three key genes in blood were identified: PYHIN1, PRSS23, and PTGDR. Another candidate biomarker, SLAMF7, was also identified via the meta-analysis method. Our findings suggest that these genes may participate in AMI pathogenesis and could be useful for the assessment of cardiovascular health in smoking people.

PYHIN1, also known as IFIX, belongs to the HIN-200 family of interferon-inducible proteins that share a 200-amino acid signature motif at their C-termini. PYHIN1 is widely expressed in immune cells and is primarily located in the nucleus [25]. It has been reported that PYHIN1 plays an important role in innate immunity. Evidence suggests that PYHIN1 can regulate pro-inflammatory cytokine induction and inflammasome activation [26, 27]. Numerous studies have found inflammasomes are associated with many adverse effects during AMI [28]. Thus, PYHIN1 may be involved in AMI pathology via affecting inflammasome activation.

PRSS23 is a newly discovered serine protease, highly conserved in vertebrates. Studies about the function of PRSS23 are rare by far. According to the record in the Human Protein Atlas [29], PRSS23 is widely expressed in all tissues, mainly located in the nucleus, but can also secrete into plasma. Interestingly, a recent study has found smoking caused changes in blood to methylation of the PRSS23 gene at several specific sites [30]. In line with their findings, our results show that smoking downregulated PRSS23 expression in blood, as DNA methylation usually results in decreased gene expression levels. Although the role of PRSS23 in AMI remains obscure, evidence showed that PRSS23 in cardiac endothelial cells may be involved in the cardioprotective effect of microRNA-532 during AMI [31].

PTGDR is a membrane receptor for prostaglandin D_2_ (PGD_2_), widely expressed in NK cells, dendritic cells, and T cells [29]. PTGDR is best known for their role in allergic diseases such as asthma [32, 33]. To date, little attention has been paid to the role of PTGDR in the cardiovascular system. However, the effect of its ligand PGD_2_ on the cardiovascular system has been reported a lot. Actually, it is well known that prostaglandins play important protective roles in cardiovascular diseases by increasing vascular permeability, thereby resisting atherosclerosis and thrombosis. Recently, it has been found that PGD_2_ is able to regulate the phenotypic conversion of vascular smooth muscle cells, which leads to neointima formation in occlusive arterial disease [34].

SLAMF7 is a signaling lymphocytic activation molecule, also known as CD319, CRACC, and CS1. SLAMF7 is a cell surface protein and is critical for the physiological functions of the NK cell [35]. It is worth noting that not only NK cells, SLAMF7 is also expressed on NK-T cells, T cells, B cells, dendritic cells, and monocytes [36]. Thus, like the above two hub genes, PYHIN1 and PTGDR, SLAMF7 may also participate in the pathophysiology of AMI by affecting Immune responses in the blood. Unfortunately, the role of SLAMF7 in AMI remains unclear currently.

Usually, it is difficult to obtain significantly over- or underexpressed genes when the sample size is relatively small. SLAMF7 is only identified by the meta-analysis method when the sample size is relatively larger. In the individual analysis, the expression of SLAMF7 was found no significant differences between AMI and control samples in the three individual AMI datasets, GSE62646 (adjust *p*-value = 0.192), GSE123342 (adjust *p*-value = 0.0548), and GSE141512 (adjust *p*-value = 0.1883). While other three hub genes, PTGDR, PYHIN1, and PRSS23, were all passed through the individual analysis, which may indicate the three genes are likely to be of great relevance to AMI as well as smoking. Meanwhile, it may also be convinced that the results of the established individual analysis, that is, intersecting DEGs obtained from individual GEO dataset, is accurate and credible.

We acknowledge the present study has several limitations. One significant limitation is the insufficient sample size for the qRT-PCR validation. We retrieved seven gene expression datasets from the GEO database, which contains 403 patients in total. While the sample size in these datasets is not small, the limited number of samples available for validation restricts our ability to draw more definitive conclusions. In addition, AMI patients are most likely smokers too, so it may be necessary to exclude current and former smokers from AMI datasets. At last, functional studies will be required to explore these hub genes in AMI, and larger studies are necessary to evaluate their roles and clinical significance of them in the increased risk of AMI due to cigarette smoking.

## 5. Conclusions

In summary, we used transcriptome analysis to identify key genes that were found both in the blood of AMI patients and people exposed to tobacco smoke. Notwithstanding some limitations, our findings are helpful for future studies and offer valuable and promising insights into the pathophysiology of AMI. More functional experiments are required to assess the three identified genes in AMI.

## Figures and Tables

**Table 1:** Major characteristics of AMI patients.

Baseline characteristics of AMI patients	
Men/women (%)	40/60
Age (years)	60 ± 13
Diabetes mellitus (%)	60
Hypertension (%)	40
ABO and Rh blood types (%)	A (20), B (40), O (40); Rh positive (100)
Hs-cTnI (pg/mL)	28,403.40 ± 26,018.46
Creatine kinase (U/L)	404.00 ± 376.20
NT-proBNP (pg/mL)	29,493.40 ± 27,191.62
hs-CRP (mg/L)	51.22 ± 82.79
Total cholesterol (mmol/L)	4.10 ± 1.08
Triglycerides (mmol/L)	1.67 ± 0.87
HDL cholesterol (mmol/L)	0.96 ± 0.17
LDL cholesterol (mmol/L)	2.67 ± 1.03
Medications	
Antiplatelet agents (%)	100
Beta-blockers (%)	60
Statins (%)	60

*Note:* Data in the table are presented as mean ± standard deviation or percentage of patients.

Abbreviations: AMI, acute myocardial infarction; HDL, high-density lipoproteins; hs-CRP, hypersensitive C-reactive protein; Hs-cTnI, high-sensitive cardiac troponin I; LDL, low-density lipoprotein; NT-proBNP, N-terminal pro-brain natriuretic peptide.

**Table 2:** The common deregulated genes identified in both AMI and smoking-related GEO datasets.

Genesymbol	AMI datasets			Smoking datasets		
	GSE62646	GSE123342	GSE141512	GSE23323	GSE23515	GSE55962
PTGDR	−0.378 (0.0245)	−0.431 (0.0225)	−0.471 (0.00784)	−0.393 (0.0004336)	−0.436 (0.01539)	−0.461 (0.0021304)

PYHIN1	−0.328 (0.0124)	−0.649 (0.00598)	−0.448 (0.00043)	−0.244 (0.0290752)	−0.484 (0.0090376)	−0.314 (0.0086557)

PRSS23	−0.724 (0.00000248)	−0.158 (0.0196)	−0.346 (0.00508)	−0.476 (0.0025599)	−0.850 (0.0092662)	−0.828 (0.0081108)

*Note:* Numbers in and out of the brackets represent the corresponding *p*-values and log_2_ FC, respectively.

Abbreviations: AMI, acute myocardial infarction; FC, fold change; GEO, Gene Expression Omnibus.

## Data Availability

The data that support the findings of this study are available in the GEO database under the accession number of GSE141512, GSE62646, GSE123342, GSE59867, GSE23323, GSE23515, and GSE55962.

## Supporting Information

Supporting Information 1 Table S1: sample information of the seven GEO datasets.

Supporting Information 2 Figure S1: volcano plot for Limma results of three AMI and three smoking-associated GEO datasets. The *x*-axis represents log_2_ FC, and the *y*-axis indicates log_10_FDR. Each dot represents a gene that had detectable expression in both groups. Red dots represent genes that are significantly expressed in the AMI (A–C) or smoking (D–F) group compared with the control. FC, fold change; FDR, false discovery rate.

Supporting Information 3 Figure S2: qRT-PCR analysis of the four hub genes. The solid black line represents the median, while the dashed black line represents the quartile. Two-way ANOVA analysis followed by Bonferroni's multiple comparisons test: *⁣*^*∗*^*p* < 0.05, *⁣*^*∗∗*^*p* < 0.01.

Supporting Information 4 Table S2: common DEGs in three AMI-associated GEO datasets. FC, fold change.

Supporting Information 5 Table S3: common DEGs in three smoking-associated GEO datasets. FC, fold change.

Supporting Information 6 Table S4: meta-analysis results. Fdr_pval: *p*-value adjusted for multiple testing by the FDR method. Zval: *Z* value is a test statistic for *Z*-tests that measure the difference between an observed statistic, the gene expression levels, and its hypothesized population parameter in units of the standard deviation.

Supporting Information 7 Table S5: GSEA results for DEGs identified by the meta-analysis method. NES, normalized enrichment score.
