# Supplementary figures and images for: Gene Expression Signatures of Smoking and Acute Myocardial Infarction: A Blood Transcriptome Analysis
Source: Mediators Inflamm. 2025 Jan 15;2025:2431090. doi: 10.1155/mi/2431090 (PMC11753852; doi:10.1155/mi/2431090)

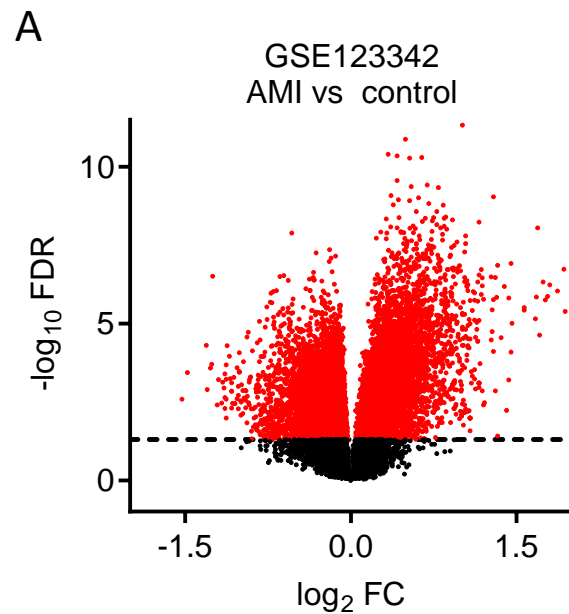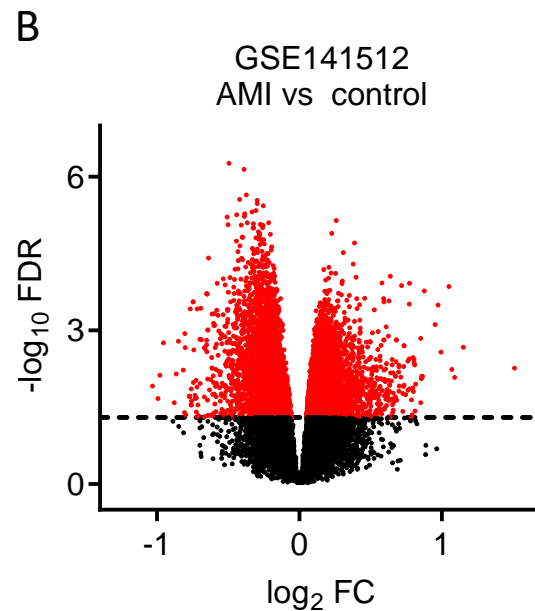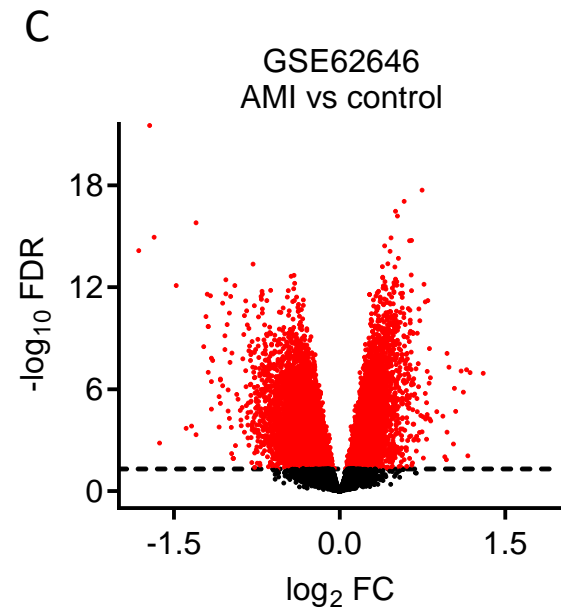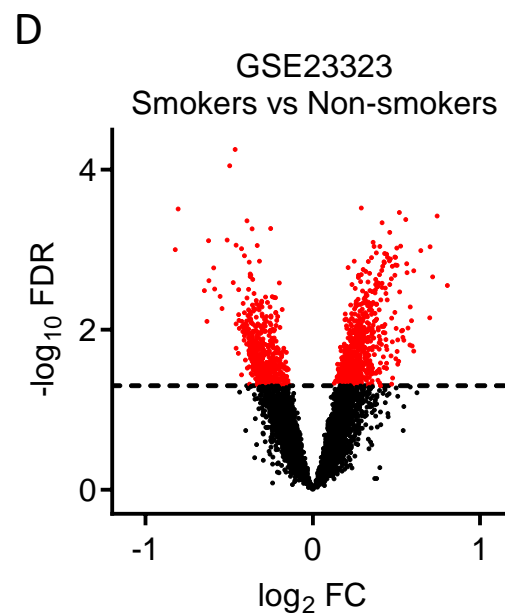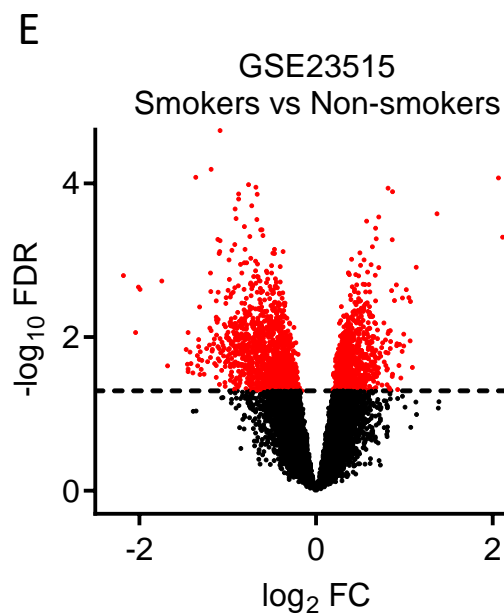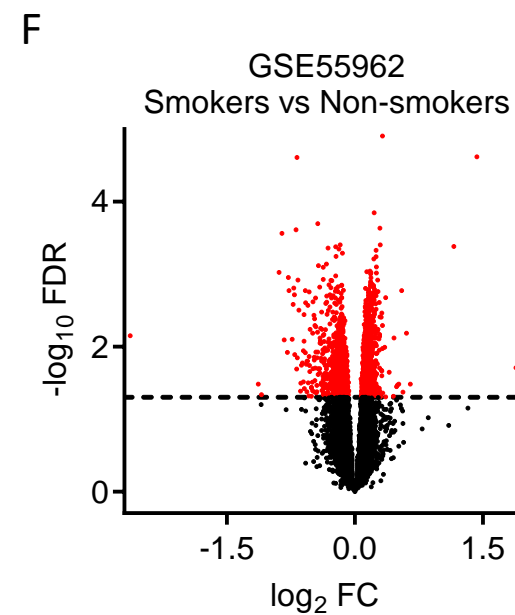

Supplement: Supporting Information 2 — Figure S1: volcano plot for Limma results of three AMI and three smoking-associated GEO datasets. The x-axis represents log2 FC, and the y-axis indicates log10FDR. Each dot represents a gene that had detectable expression in both groups. Red dots represent genes that are significantly expressed in the AMI (A–C) or smoking (D–F) group compared with the control. FC, fold change; FDR, false discovery rate. [file 2431090.f2.pdf]

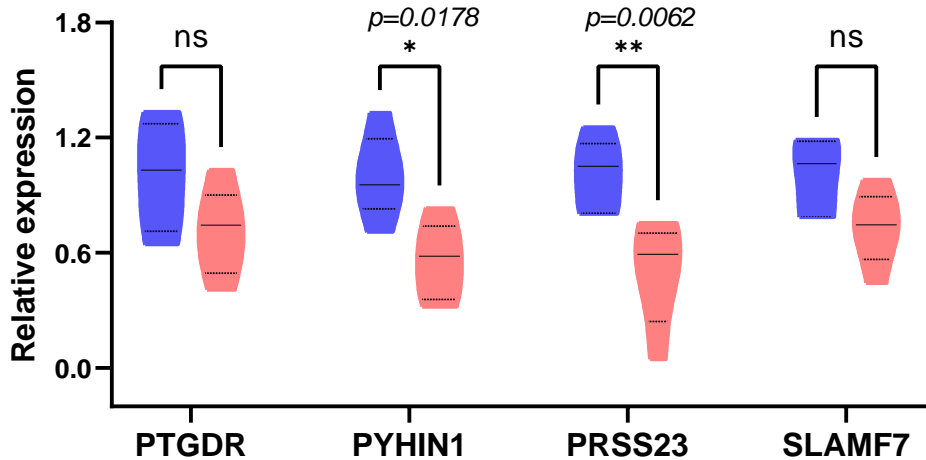

Supplement: Supporting Information 3 — Figure S2: qRT-PCR analysis of the four hub genes. The solid black line represents the median, while the dashed black line represents the quartile. Two-way ANOVA analysis followed by Bonferroni's multiple comparisons test: ⁣∗p < 0.05, ⁣∗∗p < 0.01. [file 2431090.f3.pdf]
